# GenoVault: a cloud based genomics repository

**DOI:** 10.1186/s13040-021-00268-5

**Published:** 2021-07-29

**Authors:** Sankalp Jain, Amit Saxena, Suprit Hesarur, Kirti Bhadhadhara, Neeraj Bharti, Sunitha Manjari Kasibhatla, Uddhavesh Sonavane, Rajendra Joshi

**Affiliations:** HPC-M&BA) Group, Centre for Development of Advanced Computing (C-DAC), Pune, MH, 411008 India

**Keywords:** Cloud, OpenStack, Genomics repository, NGS

## Abstract

GenoVault is a cloud-based repository for handling Next Generation Sequencing (NGS) data. It is developed using OpenStack-based private cloud with various services like keystone for authentication, cinder for block storage, neutron for networking and nova for managing compute instances for the Cloud. GenoVault uses object-based storage, which enables data to be stored as objects instead of files or blocks for faster retrieval from different distributed object nodes. Along with a web-based interface, a JavaFX-based desktop client has also been developed to meet the requirements of large file uploads that are usually seen in NGS datasets. Users can store files in their respective object-based storage areas and the metadata provided by the user during file uploads is used for querying the database. GenoVault repository is designed taking into account future needs and hence can scale both vertically and horizontally using OpenStack-based cloud features. Users have an option to make the data shareable to the public or restrict the access as private. Data security is ensured as every container is a separate entity in object-based storage architecture which is also supported by Secure File Transfer Protocol (SFTP) for data upload and download. The data is uploaded by the user in individual containers that include raw read files (fastq), processed alignment files (bam, sam, bed) and the output of variation detection (vcf). GenoVault architecture allows verification of the data in terms of integrity and authentication before making it available to collaborators as per the user’s permissions. GenoVault is useful for maintaining the organization-wide NGS data generated in various labs which is not yet published and submitted to public repositories like NCBI. GenoVault also provides support to share NGS data among the collaborating institutions. GenoVault can thus manage vast volumes of NGS data on any OpenStack-based private cloud.

## Introduction

Next-generation sequencing (NGS) platforms are producing enormous volumes of data with the introduction of high throughput technologies [1]. Nucleotide sequence data are being produced at exponential rates leading to the production of terabytes of data [2]. There is a need to store and organise such enormous data in a manner that enables easy access to data in a secure environment for the research community. Several traditional solutions are available in the public domain which use distributed DBMS over file servers [3]. Although relational databases have been primarily used in databases for many years, there are other solutions, such as Object Oriented and NoSQL for storing datasets [4]. Relational databases, although good for transactions with ACID properties [5], have challenges in handling huge volumes of data [6] due to adherence to strong schema. Security, scalability and integrity are the primary factors considered for understanding the advantages and limitations of the file-based, column-oriented design of relational database storage architecture. Depending upon the complexity of the experiment, the size of raw data generated by NGS also increases and can reach up to terabytes for a single organism and for multiple samples the size can increase up to petabytes [7]. A private cloud-based repository can help an organization to keep the data locally and also share the data among its collaborating institutes. In order to improve the ease of access to such datasets, we have developed a user-friendly platform named GenoVault for the retrieval and storage of NGS data. GenoVault is a software suite which enables cloud-based genomic repository facilitating quick archival and retrieval of NGS data. The repository is integrated with an analytical engine. GenoVault utilizes the full advantage of OpenStack based Cloud and distributed computing using commodity storage. Users can upload the genomics sequence data onto the GenoVault using web-based or JavaFX interface along with metadata which is stored in a distributed manner on the OpenStack-based cloud. GenoVault can be deployed on any OpenStack [8] based public or private cloud infrastructure.

## Need of genomics repository

There is a manifold increase in the rate and amount of Next generation sequencing (NGS) data. The size of raw data generated from NGS also increases rapidly which can be terabytes in size for a single organism. Heterogeneity in the bioinformatics data formats used by researchers for storage makes it cumbersome to share data among collaborating institutes. The generated genomic data files along with the metadata can be stored in a genomics repository. Thus, a centralized uniform repository would be of enormous importance for the researchers working in the area of genomics [9]. Collaborators belonging to different laboratories can reuse the deposited raw NGS data and employ different protocols to analyse the data. This would ensure optimal utilisation of the data generated. Researchers would benefit with an infrastructure that ensures maximum accessibility, stability and reliability to facilitate working with and sharing of research data. Biological researchers carrying out bench-work generate NGS data but may be unable to analyze the data because either there is a lack of bioinformatics expertise or unavailability of compute and storage infrastructure. With data sharing, the research data does not remain restricted to the lab where it is generated. Dealing with the genomics data requires not only managing large data volumes but also being able to deal with the many different data formats (fastq; bam; sam; bed; vcf), query types and other dynamic real-time requirements. Users need to locate, understand, analyze and visualize the data to be able to use it effectively, which in the present scenario has scope to improve as there is a lack of suitable techniques, tools and training [10]. There is an urgent need to create a genomics data infrastructure to help users to store their data and process it quickly, easily and effectively extract knowledge. The infrastructure should be supported with data mirroring and disaster recovery sites and follow a multi-tier approach to address data consistency and data redundancy issues. The physical infrastructure should be supplemented by the data access, ownership policies and security considerations. In the near future, it is expected that there would be extensive sequencing to cover the entire biodiversity across the globe (humans, plants, animals, microbes). This will lead to a tsunami of sequence data of all species, which must be secured and stored in an efficient manner [11]. The infrastructure required for creating such a repository should be of massive scale. In order to manage this vast volume of data, we need to build an advanced genomics data archival retrieval system.

## Study of existing genomics platforms

Many customized common data repositories are available to help researchers working in a collaborative manner and yield high-quality research. Institutes like National Center for Biotechnology Information (NCBI) [12], European Bioinformatics Institute (EBI) [13], DNA Data Bank of Japan (DDBJ) [14] provide an open platform for data sharing worldwide. International Nucleotide Sequence Database Collaboration (INSDC) [15], GenBank (at NCBI) [16]; ENA (at EMBL EBI) [17] and DDBJ (at NIG) [18] have been serving as nucleotide sequence repositories for researchers across the world. These repositories, apart from including raw sequence data, also provide access to alignments, assemblies and functional annotation. GenBank, EMBL and DDBJ nucleic acid sequence data banks have from their inception, used tables of sites and features to describe the roles and locations of higher-order sequence domains and elements within the genome of an organism. DDBJ provides a nucleotide sequence archive database and accompanying database tools for sequence submission, entry retrieval and annotation analysis. DDBJ is administered by the Center for Information Biology and DDBJ (CIB DDBJ) [14] of the National Institute of Genetics. The EMBL Nucleotide Sequence Database is maintained at the European Bioinformatics Institute (EBI) [19]. National Center for Biotechnology Information (NCBI) [12] resource is the most used worldwide and contains a variety of databases. GenBank database is an annotated collection of all publicly available nucleotide sequences. GenBank and other repositories receive sequences produced in laboratories throughout the world and continues to grow at an exponential rate, doubling every ten months [20]. The analysis of such large data can help researchers to improve and excel in areas like human health, livestock, agriculture and environment. Genomics England has successfully used commercial implementation of object storage (Quantum ActiveScale) for handling 10,000 genomes project and COVID-19 pandemic associated viral genomics data [21]. Many public cloud providers also provide genomics-based solutions like Google Genomics [22], AWS Genomics [23] and Microsoft Genomics [24]. Genomics data is very well suited for cloud-based storage and analytics of large-scale raw data types, which can be further analysed by scientists depending on their research requirements. Many cloud-based genomics solutions are also available like DNAnexus [25], Seven Bridges [26], DNASTAR [27], CLC Bio [28] which provide various aspects of cloud from Software-as-a-Service(SaaS) to Platform-as-a-Service(PaaS). A detailed study of many such existing cloud-based genomics platforms was conducted which helped in development of the GenoVault.

## GenoVault private cloud repository

**Fig. 1:**
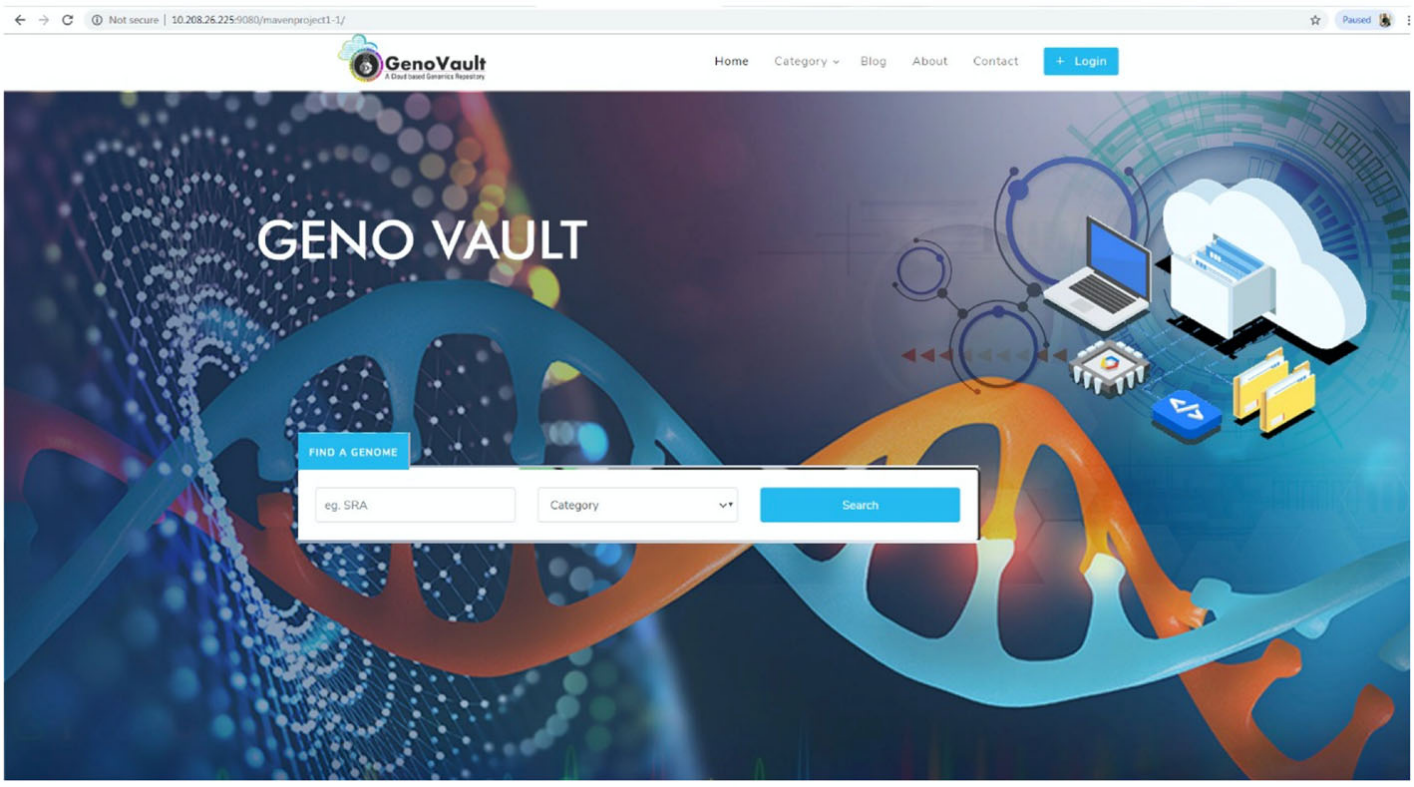
GenoVault Web Client: A user friendly GUI to access GenoVault

**Fig. 2:**
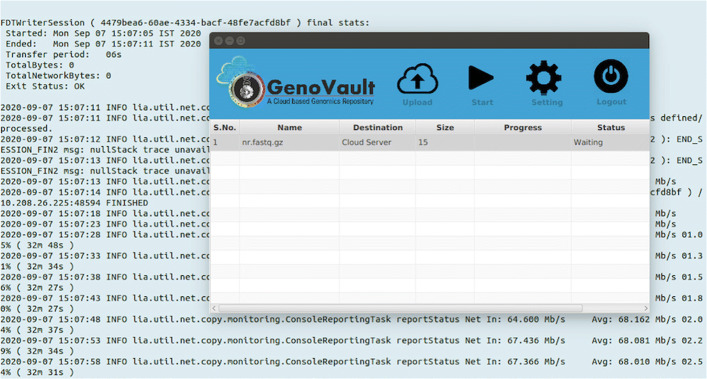
GenoVault file upload Client: A standalone application for large file upload

GenoVault is a private cloud-based data storage that can use existing OpenStack based cloud deployment [29]. GenoVault can be implemented as an organization centric or in peer to peer collaborative manner. GenoVault helps in increased participation by providing real-time access to a wide variety of genomics data with seamless integrated cloud environment. GenoVault provides security zones [30] which are the basic area of trust that can be leveraged by discretionary access control by a group of users. As genomics data is growing, the size of raw data generated also increases. In order to improve the ease of access to NGS datasets, we have developed GenoVault with a user-friendly interface for storage and retrieval of genomics data [31]. The solution is delivered in the form of a software suite along with support for analytical engines. As GenoVault is based on OpenStack [29] cloud, it exploits and utilizes the full advantage of cloud computing, distributed computing and object-based storage. Users can upload the sequence data onto the cloud using the Web or JavaFX interface of GenoVault along with metadata which is stored in a distributed manner on the cloud. The web-based GUI can be used to upload smaller files while the standalone client can be used to upload bigger files. The web client has been developed using JSF and jCloud APIs as shown in Fig. 1. OpenStack cloud, jCloud API, FDT libraries, Object storage and JSF 2.0 are used for web-interface. Standalone client enables the transfer of large data files using Fast Data Transfer (FDT) [32] as shown in Fig. 2. The standalone client has been developed using JavaFX interface and jCloud APIs.

**Fig. 3:**
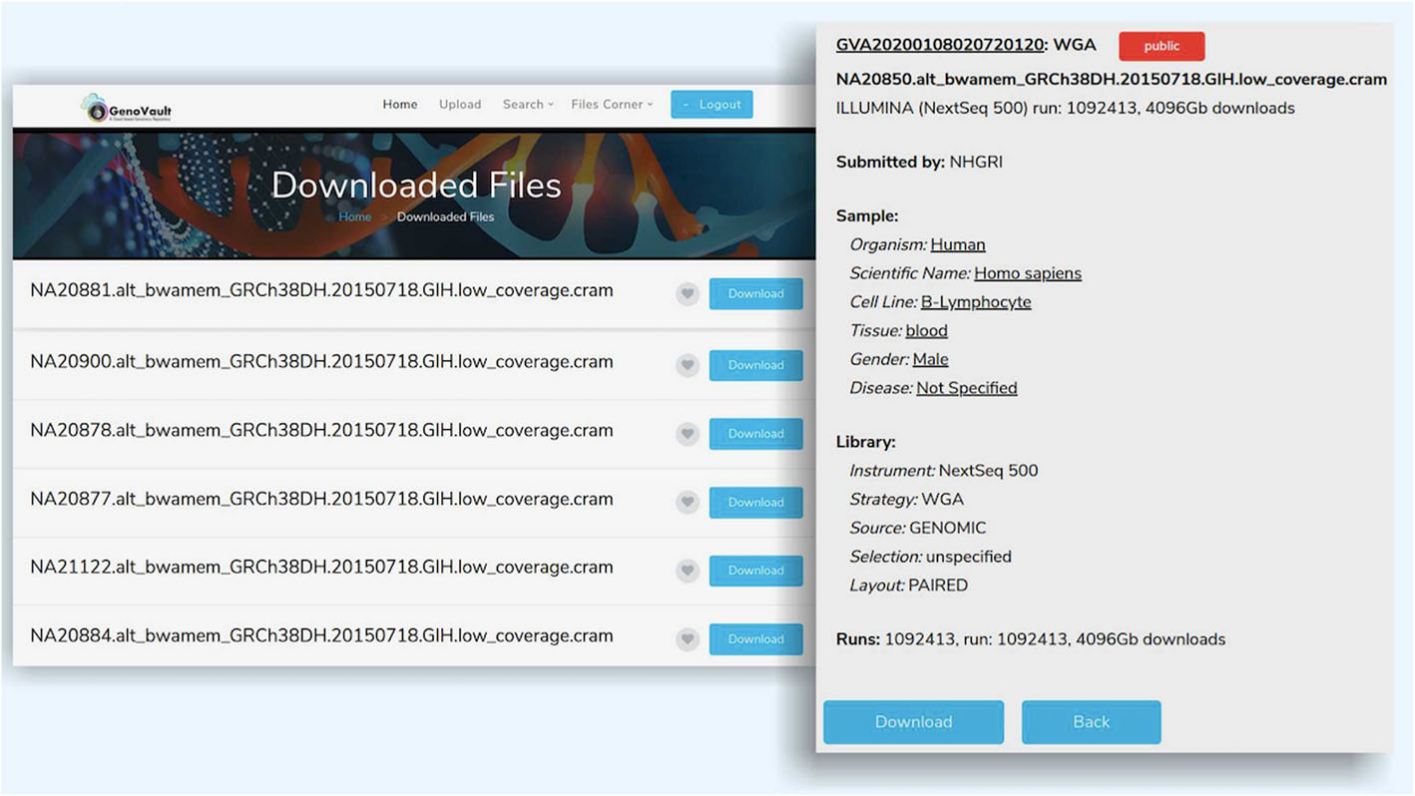
Metadata fields: Genomics data descriptor fields as object metadata

The data storage part in GenoVault is implemented using OpenStack Swift [33] which is based on a distributed object storage solution. The main advantage of object-based storage is the ability to distribute objects and their requests over a large number of commodity nodes maintaining a single namespace [34]. OpenStack key components include compute, storage (Cinder and Swift) and networking [35]. Swift offers cloud storage software that can store and retrieve data. Swift container scales and is optimized for durability, availability, concurrency across the entire data set which is used for storing unstructured data that can grow without any bounds. Users can upload files using either web-based or standalone client along with metadata [36]. Metadata contains information regarding the type of sequencing platform used for sequence generation, number of samples, source organism, etc. as shown in Fig. 3.

Uploaded files are accessible to the user and visible in the public domain. Users can search and download data if it has a flag for public access using a web-based user friendly interface. Standalone desktop client is capable of transferring files of large sizes. Users have their own storage area for uploading and downloading data. NGS data files are stored in the cloud as objects. The objects are stored in a distributed manner across Swift nodes. Distributed storage enables efficient retrieval of the genomics data.

Development was carried out using OpenStack as a back-end with various services like nova, cinder, neutron and keystone for authorization and authentication of the user. Object-based storage enables data to be stored as objects instead of files or blocks which enables faster retrieval from different distributed object nodes. These objects are stored in the container and each container is capable of storing a large number of files. GenoVault architecture allows verification of the data in terms of integrity and authentication before making it available in the public domain. GenoVault repository is designed taking into account future needs. The backend OpenStack based storage supports both horizontal and vertical scaling [37]. Horizontal scaling means scaling by adding more nodes whereas vertical scaling refers to scaling by adding more compute power (e.g. CPU, RAM) to existing nodes [38].

**Fig. 4:**
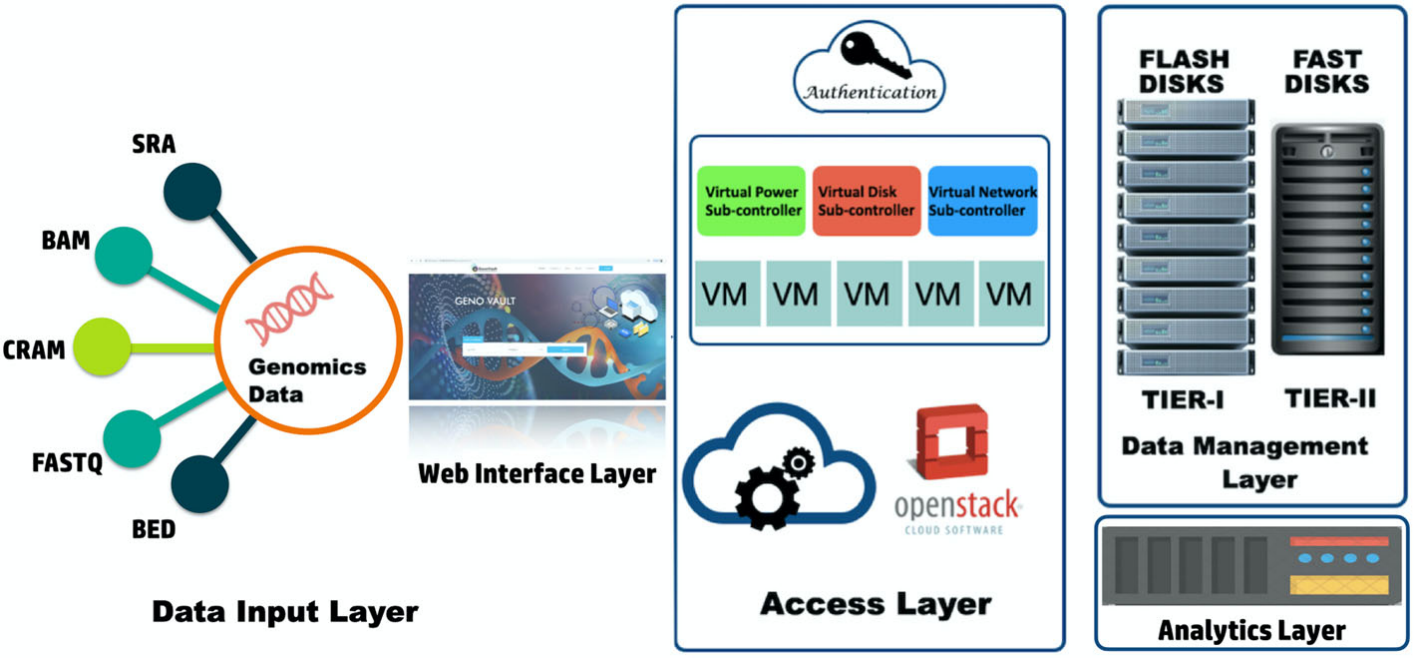
GenoVault Architecture: The system components and the sub-systems layers

The GenoVault Architecture is divided into components as shown in Fig. 4.

### Data input

Data input involves providing data from multiple heterogeneous sources like public genomics repositories and genomics data generated by various research labs. Various NGS file formats can be uploaded in GenoVault. Format validation at this step will make the retrieval operations like sorting, summarizing, consolidating, integrity checking, building indices and partitions easier. Data cleaning and data transformation are important steps in improving the quality of data and giving fast retrieval results. Provision is available in GenoVault for the validation of various file formats using standard tools like Picard sam or bam file validator [39], fastQValidator [40], gffValidator [41] and others. There is a staged upload mechanism in GenoVault. The curator can do the data validation manually and only after authorization of the curator, genomics data is uploaded in Cloud and accession number is allocated. This curation step would enable removal of any erroneous data from being uploaded in GenoVault.

### Access layer

This layer accommodates all user related interaction applications, tools, hypervisor, firewall, software packages, software-defined network (SDN), virtual machines and volume storage [42]. This layer facilitates the user to set up all access controls secured by firewalls accessing SDN to volume/storage. It is directly connected to the primary storage with high speed networks like LAN and Infiniband.

### Data management layer

This layer has a multilayer mechanism which refers to configuring data storage infrastructure as a set of tiers, where each tier comprises a collection of media (memory, disk or tape) having distinctive performance, capacity and cost characteristics.

#### Primary storage (Tier-I)

The primary storage is used for high I/O random access data storage mechanism. Primary storage is faster as compared to secondary storage due to the high-speed drives used for high retrieval rate of data. The primary storage is directly connected to a high-speed network over LAN and Infiniband.

#### Secondary storage (Tier-II)

Secondary storage devices, which are capable of storing high volume data are less expensive but slower than primary storage. In secondary storage data are written once while reads are performed by several applications. It is well connected to the application layer through high speed networks like LAN and Infiniband and conjointly with storage devices.

### Analytics layer

Extensive computational analysis using a number of algorithms and applications is required to infer scientific insights from genomics data generated by various research centres. We provide an analytics platform as shown in Fig. 4 for analysis of the genomics data using standard tools. This enables researchers to get a preliminary overview of the trends in their data and hence serve as a good starting point for more extensive analysis. The analytics infrastructure consists of a Hadoop/Spark based node [43]. The Hadoop/Spark node is deployed along with the cloud resources using Swift web-services of OpenStack. The Hadoop node denotes a fully functional hardware as well as a software stack for genomics data analysis. Hadoop consists of the Hadoop Common which provides access to the distributed file system [43]. The Hadoop Common package contains the necessary JAR files and scripts needed to start Hadoop. The package also provides source code, documentation and a contribution section which includes projects from the Hadoop Community. For effective scheduling of job, every Hadoop compatible file system should provide location awareness. Hadoop applications can use this information to run job on the node where the data is stored. The Hadoop Distributed File System (HDFS) [44] uses the information while replicating data to keep multiple copies of the data on different nodes for fault tolerance.

## Software and toolset components

There are many technologies that are available to build software stack. Various technologies and platforms are used for development of GenoVault like Java, Cloud Computing using OpenStack [29], Object Storage Swift, Web Service, Swing, Struts, JSF etc. The essential software components which are required for deployment of GenoVault are Java, MySQL, OpenStack, Swift based Object Storage Setup and Wildfly [45] or any other application server.

### Storing data

Object-based storage needs to be able to handle high capacity and provide low latency. It can be achieved by using hyper scale environments or NAS in a more traditional way. Very high-end enterprise cluster and SAN or Cloud environments with object storage are required for storing NGS data.

### Moving data

Moving data between collaborators is also non-trivial and shipping hard drives is being used due to poor internet bandwidth. A cloud-based gateway to scalable, high performance and open access analytics tools to do a run-time analysis with genomics data and convey the desired results to the community is essential. In cloud-based repositories data movement is minimized by availing the computation near data. It requires only input data and result data movement over the internet, all the intermediate data can remain in the Cloud.

## Prototype sample data upload and testing

The 1000 Genomes Project [46](1KGP) started in 2008 and completed in 2015, creating huge variation (with at least 0.01 of minimum allele frequency) and genotype data. This project was completed in four stages including the pilot phase. 1KGP includes 26 different populations, divided into five super populations namely African (AFR), Ad Mixed American (AMR), East Asian (EAS), European (EUR) and South Asian (SAS). The alignment files (in binary format) of 109 samples pertaining to Gujaratis in Houston (GIH) population were uploaded [47] into GenoVault as a prototype sample data. Data size of 109 bam files is 850 GB, wherein the smallest file size is 7.7 GB and the largest file is 28 GB. These alignment files have been obtained after reference guided assembly using genome build GRCh38. The coverage of each of these samples is in the range of 2-4 X. These files are suitable for variant discovery at cohort level. After uploading these files into GenoVault, they were stored along with their corresponding metadata like accession details, sequencing platform, gender of the sample, population details. These metadata later aid in retrieval of the subsets as per user-requirements. As shown in Fig. 2 we have observed a good performance in upload of data. An average speed of 68 Mb/s is observed while uploading the file through the FDT client. The data can be downloaded from the IGSR: The International Genome Sample Resource website [47]

## Discussion

GenoVault provides a complete infrastructure and ecosystem for the storage, management and retrieval of genomics data. GenoVault has been developed by a multidisciplinary group of researchers from genomics as well as software engineers. It is useful in carrying out research pertaining to all aspects of genomics by providing solutions to problems which arise with increasing use and handling of genomics data. Development of a large cloud-based storage infrastructure dedicated for genomic data in conjunction with tools for advanced data archival retrieval of genomic data is the need of today. GenoVault is thus an advanced centralized genomic repository useful in rapid inference of the genomics data.

## Conclusion

GenoVault is a cloud-based genomics repository in which the scientists, researchers and healthcare institutes can store and retrieve the genomics data for public or private access using cloud-based infrastructure dedicated for genomic data. A common data repository using cloud technology will help researchers to work in a collaborative manner to yield high quality research. Most of the researchers either deposit their data to the public repositories like NCBI, EBI and DDBJ or keep the data with themselves in local storage. As most of the local storage research data is not submitted in international public repositories due to formalities, procedural issues, unpublished research etc. so there are chances that after a certain duration most of the data and research are lost. GenoVault provides a local centralized repository system where researchers can store and retrieve their NGS data with ease besides supporting multi-organization collaborative research. Once the researchers are ready they can submit the published NGS data from local private cloud to the international public repositories.

## Availability of software

The software is available to download at the link: https://www.cdac.in/index.aspx?id=bio_products

The software public repository is available at the link: https://github.com/bioinformatics-cdac/GenoVault

## Abbreviations

NGS: Next Generation Sequencing
NCBI: National Center for Biotechnology Information
EBI: European Bioinformatics Institute
INSDC: International Nucleotide Sequence Database Collaboration
DDBJ: DNA Data Bank of Japan
NIG: National Institute of Genetics
DBMS: Database Management System
ACID: (atomicity, consistency, isolation, durability)
SQL: Structured Query Language
SDN: Software-defined network
LAN: Local Area Network
I/O: Input Output
NAS: Network-attached storage
SAN: Storage area network
AWS: Amazon Web Services
SFTP: Secure File Transfer Protocol
